# The application of a monolithic triphenylphosphine reagent for conducting Appel reactions in flow microreactors

**DOI:** 10.3762/bjoc.7.194

**Published:** 2011-12-08

**Authors:** Kimberley A Roper, Heiko Lange, Anastasios Polyzos, Malcolm B Berry, Ian R Baxendale, Steven V Ley

**Affiliations:** 1 Innovative Technology Centre, Department of Chemistry, University of Cambridge, Lensfield Road, Cambridge, Cambridgeshire, CB2 1EW, UK; 2 GlaxoSmithKline, Gunnels Wood Road, Stevenage, Hertfordshire, SG1 2NY, UK

**Keywords:** Appel reaction, bromination, flow chemistry, solid-supported reagent, triphenylphosphine monolith

## Abstract

Herein we describe the application of a monolithic triphenylphosphine reagent to the Appel reaction in flow-chemistry processing, to generate various brominated products with high purity and in excellent yields, and with no requirement for further off-line purification.

## Introduction

Flow chemistry is well-established as a useful addition to the toolbox of the modern research chemist, with advantages accrued through increased efficiency, reproducibility and reaction safety [1–6]. Further benefits can be realised when flow processing techniques are combined with the use of solid-supported reagents and scavengers, which allow telescoping of reactions or in-line removal of byproducts to both increase the purity profile of the output product stream and to, ideally, negate the need for subsequent purification [7–12]. Reagents on macroporous or gel-type beads are commonly used; however, these can suffer from poor mass transfer as well as presenting practical problems caused by the swelling or compression characteristics of the beads related to the solvent employed. To circumvent some of the issues with bead-type supports, monoliths have been developed as replacements for use in continuous-flow synthesis. Monoliths are a single continuous piece of uniformly porous material prepared by precipitation polymerisation of a functionalised monomer [13–17]. They have been shown to have superior chemical efficiency over other bead-based materials, due to enhanced mass transfer governed by convective flow rather than diffusion, as well as possessing lower void volumes [18]. Practically, their rigid structure is maintained over a wide range of solvents and under reasonable pressure due to the high degree of cross linking, making them easier to use in flow processes. Historically monoliths have traditionally been used to facilitate the isocratic separation of peptides [19]; however, our group and others have shown interest in using monolithic supports to facilitate key chemical transformations [20–31]. We recently reported on the development of a new monolithic triphenylphosphine reagent and its use in the Staudinger aza-Wittig reaction in flow [32–33]. Here we discuss the application of this monolith to the transformation of an alkyl alcohol into the corresponding alkyl bromide by using carbon tetrabromide in the Appel reaction.

**Scheme 1:**
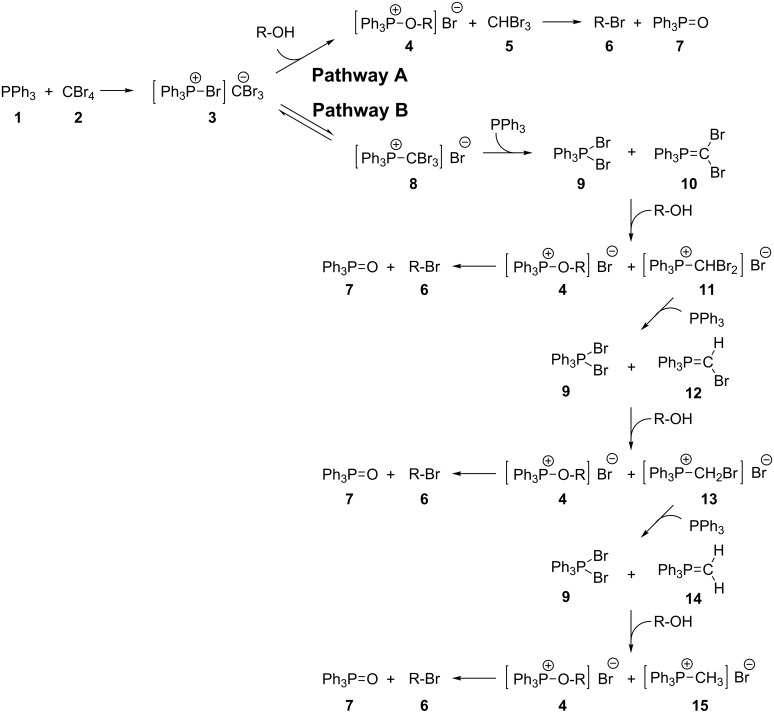
The two proposed mechanistic pathways for the Appel reaction.

In the 1960s Ramirez and co-workers reported the formation of a phosphine–methylene species when triphenylphosphine was mixed with carbon tetrabromide [34]. This was utilised by Appel in the mid 70s, who reported on the use of triphenylphosphine and carbon tetrachloride to convert an alcohol into the corresponding alkyl chloride [35]. The reaction produces byproducts, such as triphenylphosphine oxide, during the reaction that can be very difficult to remove, and extensive, time-consuming purification protocols are often needed in order to isolate the desired product in high purity. We envisioned that the use of an immobilised triphenylphosphine source in combination with continuous-flow technologies could circumvent this problem, allowing easy separation of the phosphine side products through its retention on the solid phase. Examination of the literature revealed that a variety of different bead supports have been used to facilitate this reaction to produce chloro-, bromo- and iodoalkanes from the corresponding alcohols [36–38]. However, the flow characteristics of beads make these techniques undesirable for application in a continuous-flow setup, and the relatively high cost of these reagents limits their widespread use in common laboratory practices. It is particularly interesting that these past investigations have noted an increase in the rate of the reaction on a solid-supported reagent, attributed to neighbouring group participation as a consequence of using a polymeric source of triphenylphosphine [39]. Mechanistically, the Appel reaction has been proposed to proceed via two complex and competing pathways (Scheme 1). In pathway A the reaction is thought to proceed through the simple ion pair **3** formed by the reaction of one equivalent of triphenylphosphine (**1**) and one equivalent of carbon tetrabromide (**2**). This can react with the alcohol substrate to form an oxy-phosphonium **4** along with bromoform (**5**), which is removed under reduced pressure at the end of the reaction along with the solvent. The oxy-phosphonium salt **4** then reacts with the bromide counterion to produce the substituted product **6** along with the triphenylphosphine oxide byproduct (**7**). However, **3** is in equilibrium with the inverted ion pair **8**, which can proceed via pathway B in which **8** reacts with a second equivalent of triphenylphosphine to form the dibromophosphorane **9** and phosphorane **10**. This dibromophosphorane **9** is then able to react with an alcohol to give intermediate **4**, which can proceed to the product, whereas **10** proceeds to a phosphonium salt intermediate **11**. This can then react again with another equivalent of triphenylphosphine and continue reacting in a similar manner until the methylphosphonium salt **15** is formed. This pathway, therefore, can use up to four equivalents of triphenylphosphine to give up to three brominated products. The rate determining step for both pathways has been proposed to be the formation of an active halogenating species **3** or **10** [40]. The increase in the rate of reaction with regards to the Appel reaction, with solid-supported triphenylphosphine compared to the solution-phase counterpart, is proposed to be a result of neighbouring-group participation assisting in the formation of the active species **9** and **10** in pathway B [39,41–42]. Analysis by gas chromatography of chloride Appel reactions indicated that the relative proportion of chloroform was a lot lower than would be expected if both pathways were followed equally in both the solution or solid-phase reactions (5% with solution-based triphenylphosphine and 18–29% with solid-supported triphenylphosphine), indicating that path B is the major pathway in either case [40,43].

Using a polystyrene-based triphenylphosphine monolith we hoped to benefit from the accelerated rate of reaction observed as well as to circumvent problems associated with the use of bead-based immobilised reagents in continuous flow.

## Results and Discussion

### Formation of the triphenylphosphine monolith

The triphenylphosphine monoliths were formed by precipitation polymerisation of the appropriate phosphine monomer with a cross-linking component and a porogen [32–33]. A stock solution of the functionalised monomer (diphenyl(4-vinylphenyl)phosphine), cross-linking material (divinylbenzene and styrene) and porogen (1-dodecanol) was heated to 50 °C until a homogeneous solution was obtained. The dibenzoyl peroxide was then added and the mixture maintained at elevated temperature (50 °C) until the initiator had dissolved (approximately 5 minutes). The mixture was decanted into a glass column and the ends were sealed with custom-made PTFE end pieces. The column was incubated at 92 °C [44–45] for 48 hours in a Vapourtec R4 heater to give a white polymeric solid, which filled the column. It was noted that the addition of styrene as part of the cross-linking component was necessary to increase the active loading of the monolith during the reactions. Dibenzoyl peroxide was chosen as a radical initiator as it was found to be soluble in the polymerisation mixture at the stock solution temperature of 50 °C, giving a homogeneous mixture. The slower initiation rate compared to azo-based initiators also ensured the entire polymerisation mixture was at the target temperature before precipitation of polymer chains occurred (approximately one hour after heating at 92 °C), ensuring a more homogeneous polymerisation. Following this polymerisation procedure, the monolith was cooled to room temperature and the end plugs were replaced with standard flow-through connectors. Dry dichloromethane was pumped through the column, which was heated to 60 °C, to elute the porogen and any unreacted monomer starting material. It was found that this polymerisation technique gave consistent, low pressure drops across the monolith, and these were consistent across multiple batches of monolith syntheses, making them ideal for use in a flow-chemistry setup. Elemental analysis showed an approximate loading of 1.87 mmol of phosphorus per gram, giving a calculated loading of 4.68 mmol of phosphorus per monolith, which is comparable to commercially available triphenylphosphine resins.

### Loading the monolith

**Scheme 2:**
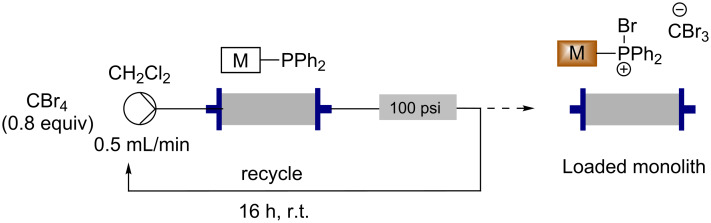
Functionalisation of the triphenylphosphine monolith by using carbon tetrabromide in a recycling process.

**Figure 1:**
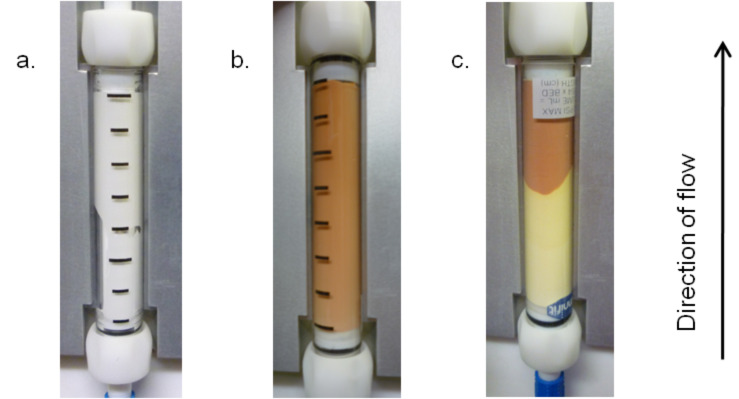
a. Unfunctionalised triphenylphosphine monolith; b. Monolith after fuctionalisation with carbon tetrabromide; c. Monolith after partial consumption of the active brominating agent.

The monolith was then loaded with carbon tetrabromide to give the active species with which to perform the Appel bromination reaction. To achieve this, carbon tetrabromide in dichloromethane [46] was recirculated through the monolith for 16 hours at room temperature (Scheme 2), resulting in a colour change from white (a) to a light brown colour (b) (shown in Figure 1). Elemental analysis revealed that the monolith consisted of 27.6% bromine showing that the carbon tetrabromide had loaded onto the monolith and there was an average of less than one molecule of carbon tetrabromide per phosphorus atom. This suggests that a complex mixture of phosphorus species is present within the monolith. Triphenylphosphine oxide, from the starting material, and unreacted triphenylphosphine, due to inaccessible sites within the monolith, are probably present along with potentially a complex combination of active brominating species. If the monolith reacts in the way reported in previous literature, then both mechanistic pathways are followed and therefore many different active brominating species are present (**3**, **8**, **9** and **10**). Although it is thought that a complex mixture is present, the loaded brominating monolith is represented as intermediate **3** for simplicity in the schemes that follow.

Reaction of this monolith with an alcohol resulted in a further colour change from a brown monolith (b) to a depleted monolith (c) (Figure 1), with a pale yellow region corresponding to the triphenylphosphine oxide, or potentially later intermediates in pathway B, formed in the Appel reaction. This same colour change was initially observed during the loading protocol when approximately one-fifth of the coloured region of the monolith was transformed to the pale yellow colour. In this case, analysis of the recycled solvent suggested the presence of 1-bromododecane and that the dodecanol porogen was not being fully removed during the initial flushing procedure, but instead was being brominated during the subsequent loading process. Unfortunately attempts to adapt the solvent system to ensure complete dodecanol removal before loading were unsuccessful. Scavenging the dodecanol in the loading process by means of a column of polymer-supported tosyl chloride or by adding calcium chloride to the recycling carbon tetrabromide solution was also unsuccessful. Although the presence of dodecanol caused a partially depleted monolith during the loading process, these monoliths were still successfully used for the Appel reaction. No detrimental effect on the reaction products was observed, although obviously a lower active loading of the monolith was observed. In order to achieve the maximum active loading of 1.3 mmol for a monolith, a previously loaded monolith was placed in-line with the unloaded triphenylphosphine monolith in the recycling procedure with carbon tetrabromide.

### Bromination reactions in flow with the loaded triphenylphosphine monolith

**Scheme 3:**
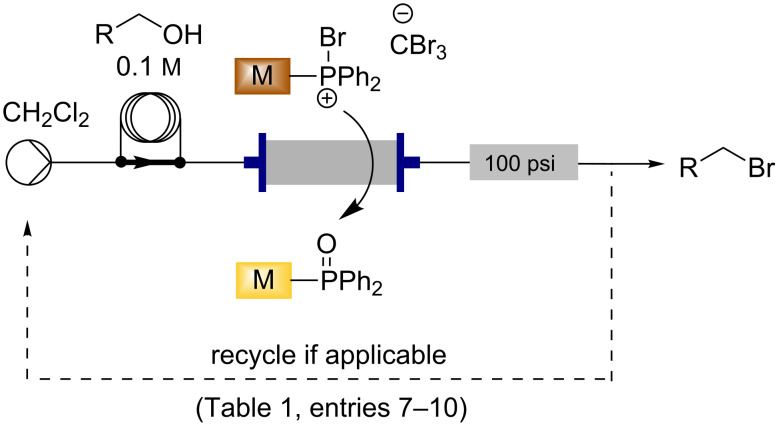
Flow synthesis of bromides from alcohols by using the functionalised triphenylphosphine monolith.

With the functionalised, active brominating monolith in hand, the transformation of an alcohol into the corresponding bromide in flow was investigated. By employing the commercially available Uniqsis FlowSyn, a 0.1 M solution of the alcohol in dry dichloromethane was prepared and loaded into the sample loop (2 mL). This solution was then switched in-line to be pumped through the monolith at a flow rate of 0.5 mL/min, the output stream was collected for 1 hour and the solvent removed (Scheme 3). For benzylic and sterically unhindered alcohols (Table 1, entries 1–6), complete conversion was achieved by a single pass of the alcohol through the monolith, requiring only solvent removal to yield the pure brominated product in high yield. An analogous reaction with polymer-supported triphenylphosphine beads, loaded and reacted in an identical way, only gave 26% conversion to halogenated material, which was impure by ^1^H NMR for cinnamyl bromide (Table 1, entry 2). For less-activated substrates, such as the iodo-substituted benzylic alcohols (Table 1, entries 7 and 8), a single pass gave incomplete conversion to the bromide, resulting in a mixture of the starting alcohol and the bromide product upon removal of the solvent. Conversion to the bromide could be increased by decreasing the flow rate; however, to obtain complete conversion it was found to be necessary to recycle the flow stream through the monolith. When a recycling protocol was employed, upon full consumption of the starting material by thin-layer chromatography the input was changed to a fresh solution of dichloromethane. The system was then flushed for a further 45 minutes at 0.5 mL/min to yield the pure bromide product following removal of the solvent. An investigation of the substrate scope revealed that starting materials containing unprotected amines could not be transformed into the corresponding bromides. Little or no mass return was observed suggesting that an aminophosphonium species was formed on the monolith in accordance with similar reactions between triphenylphosphine, bromine and amines [47]. It was possible to brominate the monoprotected aniline (Table 1, entry 9) by using the triphenylphosphine monolith, but recycling for a longer time was required, and resulted in a lower isolated yield than most of the other substrates. For the nonactivated alkyl alcohol (Table 1, entry 10), recycling for 14 hours was found to be necessary to achieve complete conversion to the desired bromide. While in this work the benzylic brominated products were not used in subsequent flow reactions, such as alkylations, these processes have been reported by us [48] and others [49] using continuous-flow technologies.

**Table 1:** Bromides prepared from the corresponding alcohols by using the functionalised triphenylphosphine monolith.

Entry	Starting material	Product	Conversion after one pass (%)^a^	Time required for full conversion^b^	Isolated yield^c^ (%)

1	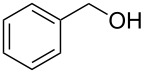	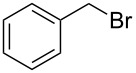	100	–	80^d^
2	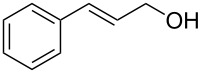	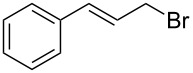	100	–	82
3	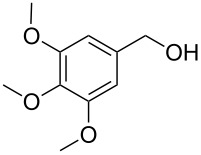	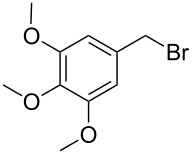	100	–	92
4	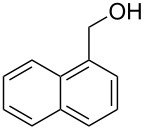	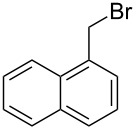	100	–	74
5	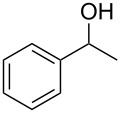	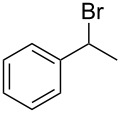	100	–	92
6	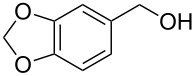	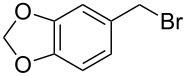	100	–	91
7	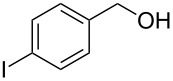	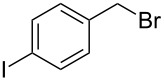	94	1 h 15 min	95
8	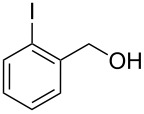	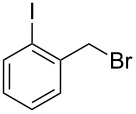	84	1 h 15 min	95
9	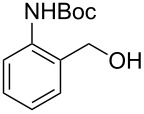	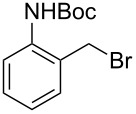	85	2 h 30 min	68^e^
10	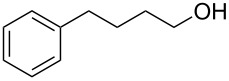	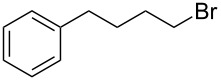	<0.5	14 h	77

^a^One pass through the monolith at 0.5 mL/min, percentage conversion determined by ^1^H NMR analysis, ^b^substrates recirculated through the monolith at 0.5 mL/min until full consumption of starting material indicated by TLC, ^c^reactions performed on a 0.2 mmol scale, ^d^volatile product, ^e^corresponding solution-phase triphenylphosphine batch process yielded 52% pure product after chromatography.

**Figure 2:**
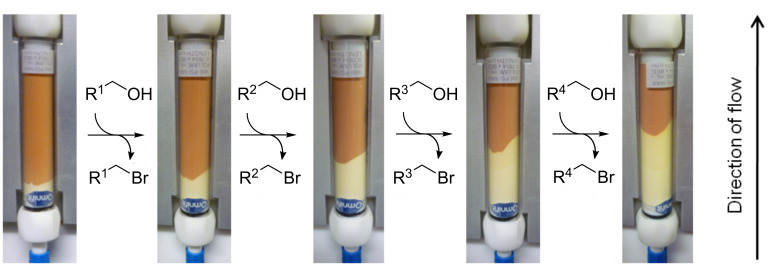
Linear decrease of the brown decolourisation.

A single monolith can be used for many different alcohols with no cross-contamination detected by ^1^H NMR between substrates. It was found that the colour of the monolith can be used as an approximate indication of the degree of loading of active brominating species on the monolith, and the off-white, pale yellow region of triphenylphosphine oxide, or later stage intermediate, was formed in a proportional manner to the quantity of the substrate that was passed through the monolith (Figure 2). Elemental analysis revealed that the off-white region contained 19.8% bromine, implying that although bromine is consumed proportionally with the consumption of the dark-coloured areas of the monolith, there is nevertheless still bromine left on the monolith. This bromine could be present in hard-to-access active sites within the polymeric structure of the monolith, but could also be accounted for by some of the late intermediates in pathway B (**10**–**15** in Scheme 1). The conversion of a particular substrate did not decrease with continued use of the monolith until the monolith became completely pale yellow, at which point the activity of the monolith decreased sharply. Up to this point the conversion of a substrate was comparable at the beginning and at the end of the monolith use. After the brown colouration had been diminished through bromination reactions, the monolith activity reduced significantly, giving 19% conversion to halogenated product by using a previously readily converted substrate (Table 1, entry 6). Each monolith was found to convert 1.3 mmol of alcohol before the conversion dropped.

It was found that heating the monolith increased the rate of reaction and thus could be used to promote the reaction of the aniline substrate (Table 1, entry 9) in one pass, albeit with a lower isolated yield [50]. While investigating the feasibility of heating the monolith in order to drive the less activated 4-iodobenzyl alcohol (Table 1, entry 7) to completion, it was found that heating the monolith resulted in the production of an impurity, at 29.7% conversion by ^1^H NMR (with 64.5% conversion to the bromide). This was identified as 1-(chloromethyl)-4-iodobenzene, i.e., the starting material underwent chlorination rather than bromination. The two halogenated products could not be separated by flash column chromatography. Interestingly this impurity was also observed in small amounts when a loaded monolith was stored for prolonged periods at room temperature in the presence of dichloromethane. The Appel reaction using 4-iodobenzyl alcohol (Table 1, entry 7) was repeated with a loaded monolith stored in dichloromethane at room temperature for 9 days and resulted in a conversion of less than 3% to the chloride by ^1^H NMR. The conversion to the chloride was found to increase with increased flushing of dichloromethane through the monolith, implying that the source of the chloride was through exchange with the dichloromethane solvent. From our initial screens, dichloromethane was found to be the optimum solvent for monolith loading and the bromination reactions. As this impurity was only observed at elevated temperatures or with monoliths that had been stored over long periods of time, we feel that we have demonstrated that it is not a significant problem for the main aim of the reagent, which is to convert alcohols into bromides without the need for purification.

## Conclusion

In summary, the monolithic triphenylphosphine reagent recently developed by our group [32–33] was also successfully applied to facilitate the Appel reaction. An active brominating species was formed readily by recirculating carbon tetrabromide through the monolith. This loaded monolith was then successfully applied to the Appel reaction, allowing the synthesis of the corresponding brominated product from an alcohol in high purity and yield, with only a simple removal of the solvent required. Activated benzylic alcohols were transformed to the desired bromide with a single pass, whereas sterically hindered or alkyl alcohols required a recycling process through the monolith to reach complete conversion to the brominated product. Heating the monolith in an attempt to accelerate the reaction was found to be unsuccessful due to the decomposition of the active brominating salt, resulting in the formation of an undesired chloride byproduct.

## Supporting Information

Supporting information features full experimental details and data for the reactions performed above.

File 1 Experimental details.
