# Supplementary material for: The application of a monolithic triphenylphosphine reagent for conducting Appel reactions in flow microreactors
Source: Beilstein J Org Chem. 2011 Dec 8;7:1648–55. doi: 10.3762/bjoc.7.194 (PMC3252869; doi:10.3762/bjoc.7.194)
Supplement: File 1 — Experimental details. [file Beilstein_J_Org_Chem-07-1648-s001.pdf]

Supporting Information

for

The application of a monolithic triphenylphosphine reagent for conducting Appel reactions in flow microreactors

Kimberley A. Roper[1], Heiko Lange[1], Anastasios Polyzos[1], Malcolm B. Berry[2], Ian R. Baxendale[1] and Steven. V. Ley[1*]

Address: [1]Innovative Technology Centre, Department of Chemistry, University of Cambridge, Lensfield Road, Cambridge, Cambridgeshire, CB2 1EW, UK and [2]GlaxoSmithKline, Gunnels Wood Road, Stevenage, Hertfordshire, SG1 2NY, UK

Email: Steven V. Ley - svl1000@cam.ac.uk

* Corresponding author

Experimental details

List of Contents

General Information S2

Formation of the triphenylphosphine monolith S4

Functionalising the monolith with carbon tetrabromide S5

Flow synthesis of bromides S6

References S10

General information

Unless otherwise specified, reagents were obtained from commercial sources and used without further purification. Divinylbenzene (80% technical grade) was obtained from Sigma-Aldrich, and diphenyl(4-vinylphenyl)phosphine (90% purity) was obtained from Hokko Chemical Industry Co., Ltd. All reactions were performed under an atmosphere of air unless otherwise specified.

Solvents were obtained from Fisher Scientific unless otherwise specified. Dichloromethane (technical grade) was passed through an activated alumina column followed by a catalytic copper purification column under an atmosphere of dry argon [1] .

Analytical thin-layer chromatography was performed on precoated glass-backed plates (Merck Kieselgel 60 F_{254}) and visualised by ultraviolet irradiation (254 nm) or by staining with aqueous acidic ammonium molybdate(IV) as appropriate.

^{1}H NMR spectra were recorded on a Bruker DPX-600 (600 MHz) spectrometer with the deuterated solvent as an internal deuterium lock and with the residual monoprotic solvent (7.26 ppm $CHCl_3$) [2] used as the internal reference. The multiplicity of a signal is indicated as: s–singlet, d–doublet, t–triplet, m–multiplet, br–broad, app–apparent or combinations of these. Coupling constants (*J*) are recorded to the nearest 0.1 Hz.

^{13}C NMR spectra were recorded on a Bruker DRX-600 (150 MHz) spectrometer with broadband proton decoupling with the deuterated solvent as internal deuterium lock and with the deuterated solvent (77.16 ppm $CDCl_3$) [2] used as an internal reference.

DEPT 135, COSY, HMQC and HMBC spectra were used to aid in the assignment of signals in ^{1}H and ^{13}C NMR spectra where appropriate.

Gas chromatography-mass spectrometry (GCMS) was performed on a PerkinElmer Turbomass Autosystem XL with a Supelco SLB™-5ms column (30 m × 0.25 mm × 0.25 µm film thickness) and positive electron ionisation (EI^+). Run parameters: injector temperature = 200 °C, injection volume = 0.5 µL, split ratio 10:1, gas = He, flow rate = 1 mL/min, start temperature = 80 °C (hold 5 min), ramp = 10 °C/min, end temperature = 240 °C (hold 1 min), total run time = 22 min. Retention time is reported as Rt. Masses are reported followed by the assignment and relative intensity in parentheses.

IR spectra were recorded on a PerkinElmer Spectrum One FT-IR spectrometer by using universal ATR sampling accessories. The samples were prepared as thin films unless otherwise specified. Letters in parentheses refer to relative absorbance with respect to the most intense peak: w – weak (<40%), m – medium (40–75%), s – strong (>75%).

High resolution mass spectrometry (HRMS) was performed on a Bruker BioApex II 4.7e FTICR utilising a positive electron ionisation (EI^+) source equipped with a direct

insertion probe. The mass reported is that containing the most abundant isotope (^{79}Br). Limit: ±5 ppm.

Elemental composition microanalysis was performed by the Microanalytical Laboratories at the Department of Chemistry, University of Cambridge and results are reported to one decimal place.

Monolithic reagents were formed and used in glass Omnifit® columns, sealed for polymerisation with custom made PTFE end pieces, heated by using a Vaportec R4 system and otherwise connected to the flow system with standard tubing connectors.

Formation of the triphenylphosphine monolith

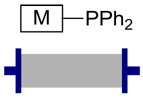

The reaction scale specified below forms a monolith that fills a 7.845 mL glass Omnifit® column (inner diameter 10 mm × 10 cm). Multiple monoliths, or monoliths of other volumes, can be made from the same stock solution by multiplying the values below as appropriate.

Divinylbenzene (0.99 g, 7.6 mmol) and styrene (0.20 g, 1.9 mmol) were added to a mixture of diphenyl(4-vinylphenyl)phosphine (1.59 g, 5.5 mmol) in 1-dodecanol (2.14 g, 11.5 mmol) and the mixture was heated to 50 °C. When complete dissolution was achieved dibenzoyl peroxide (33 mg, 0.1375 mmol, 1.45 mol % with respect to the monomer + crosslinker) was added and the mixture stirred for another 5 minutes at elevated temperature. A 10 mm inner diameter × 10 cm length glass column was filled to 7 cm height with this solution, and both ends of the column were sealed with custom-made PTFE end plugs. The column was heated at 92 °C for 48 hours by using a Vapourtec R4 convection heater, resulting in a rigid white monolith (the start of precipitation polymerisation was observed after 1 hour at temperature). Following polymerisation, the monolith was allowed to cool to room temperature, and the end seals were exchanged for standard tubing connectors. To remove the porogen and any residual nonpolymeric material, the monolith was then heated to 60 °C and washed with dry dichloromethane at 0.2 mL/min, without a back-pressure regulator, for 1 h by using the Uniqsis FlowSyn flow system. A 100 psi back-pressure regulator was added to the system and the monolith was then flushed at 0.2 mL/min for another hour. The flow rate was then slowly increased to 1.5 mL/min over the next hour, the monolith inverted and then the process repeated. The monolith was finally flushed at 0.2 mL/min for 30 minutes while being allowed to cool to room temperature.

Elemental analysis found: C, 86.9; H, 6.7; P, 5.8% (Loading = 1.87 mmol P per g monolith); Dry weight = 2.5 g; IR (thin film) ν: 3050.5 (w), 2922.7 (w), 1599.4 (w), 1480.6 (w), 1434.5 (w), 1404.7 (w), 1184.3 (w), 1118.6 (w), 1092.2 (w), 1027.3 (w), 1017.0 (w), 989.2 (w), 904.2 (w), 825.1 (w), 797.4 (w), 742.0 (m), 695.0 (s) cm^{-1}.

Functionalising the monolith with carbon tetrabromide

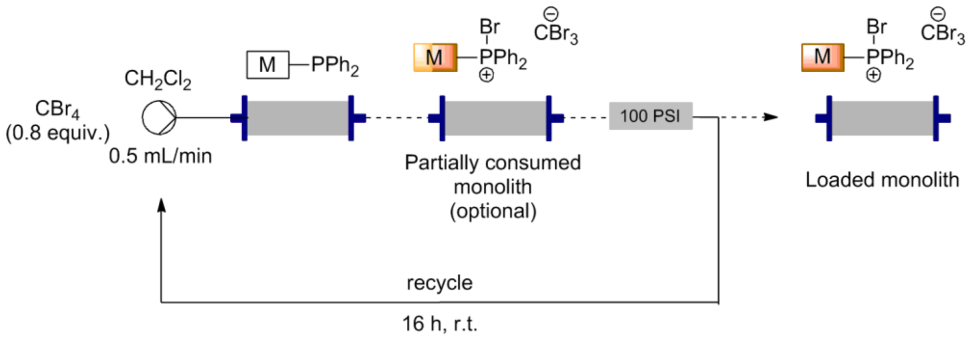

Carbon tetrabromide (1.50 g, 4.5 mmol) was dissolved in 40 mL dry dichloromethane (0.11 M) and recirculated for 16 hours under argon at 0.5 mL/min through the triphenylphosphine monolith at room temperature. The system was pressurised by using a 100 psi back-pressure regulator inline after the monolith. The monolith became decolourised during the loading process, resulting in a dark brown monolith, and an overall rise in pressure of approximately 1 bar was observed. The system was then flushed for 1 hour at 0.5 mL/min with a fresh solution of dry dichloromethane. Removal of the solvent in vacuo revealed that approximately 0.75 g carbon tetrabromide could be isolated; indicating that 0.75 g (2.3 mmol) had been consumed during the loading process.

It was noted that residual dodecanol in the monolith reacted with the active species being formed, consuming some of the loaded monolith in the above process. To prevent this, a partially used monolith was placed in sequence with the unloaded monolith in the recycling method above.

Elemental analysis found: C, 62.9; H, 5.0; P, 4.2; Br, 27.6% (number of equivalents of Br to P = 2.55:1); Dry weight = 2.5 g; IR (thin film) ν: 3658.1 (w), 2988.3 (m), 2901.4 (m), 1600.0 (w), 1485.0 (w), 1437.6 (m), 1407.3 (m), 1119.5 (s), 1066.6 (s), 827.6 (w), 796.6 (w), 750.1 (m), 722.3 (m), 703.4 (s) cm^{-1}.

Flow synthesis of bromides

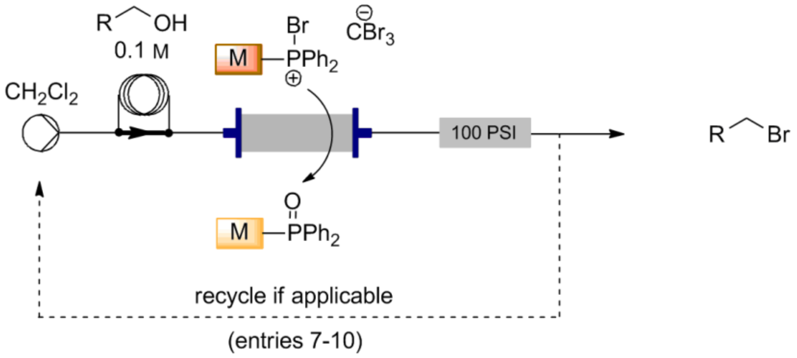

A 2 mL solution of the desired alcohol (0.2 mmol, 0.1 M) in dry dichloromethane was prepared and injected into the sample loop. This sample loop was then switched in-line and pumped at 0.5 mL/min, with a stream of dry dichloromethane under argon, through the loaded monolith prepared above and then through a 100 psi back-pressure regulator. The output stream was collected for 1 hour and then concentrated in vacuo to yield the product. If incomplete conversion was observed by ^{1}H NMR then a recycling protocol was employed for that substrate. For the substrates that required recycling, the above method was followed, but the output was directed back into the dichloromethane stock solution. When no more starting material was observed by thin-layer chromatography of the stock solution, the monolith was flushed for 45 minutes at 0.5 mL/min with a clean solution of dichloromethane. The washings from this flushing step were combined with the previous stock solution and concentrated in vacuo to yield the product.

During the synthesis of the bromides it was observed that the monolith changed colour from a dark brown colour (observed in the loading step) to an off-white, pale yellow colour. Analysis of the pale yellow region gave the data below.

Elemental analysis found: C, 64.4; H, 5.2; P, 4.5; Br, 25.9% (number of equivalents of Br to P = 2.23:1); Dry weight = 2.5 g; IR (thin film) ν: 2988.2 (w), 2913.1 (w), 1595.1 (w), 1485.8 (w), 1437.5 (m), 1407.2 (w), 1119.7 (s), 1067.0 (m), 997.8 (w), 903.2 (w), 827.2 (w), 797.9 (w), 749.9 (m), 722.2 (m), 703.5 (s), 693.9 (s) cm^{-1}.

All yields below are for a single pass through a monolith at 0.5 mL/min unless specified otherwise.

(Bromomethyl)benzene (Entry 1) [3]

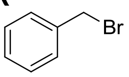

Isolated as a pale yellow oil (27.4 mg, 80% yield); ^{1}H NMR (600 MHz, $CDCl_3$) δ_H 7.39 (d, J = 7.3 Hz, 2H), 7.35 (app t, J = 7.4 Hz, 2H), 7.30 (t, J = 7.2 Hz, 1H), 4.51 (s, 2H); ^{13}C NMR (150 MHz, $CDCl_3$) δ_C 137.8 (*C*), 129.0 (2 × *C*H), 128.8 (2 × *C*H), 128.4 (*C*H), 33.5 (*C*H_2); IR (thin film) ν: 3063.3 (w), 3031.0 (w), 1495.5 (w), 1454.2 (w), 1225.8 (m), 1200.7 (w), 1068.4 (w), 1028.8 (w), 916.7 (w), 869.2 (w), 812.7 (w), 801.7 (w), 756.0 (m), 692.1 (s), 667.0 (w) cm^{-1}.

(*E*)-(3-Bromoprop-1-en-1-yl)benzene (Entry 2) [4]

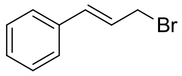

Isolated as a pale yellow oil (32.6 mg, 82% yield); ^{1}H NMR (600 MHz, $CDCl_3$) δ_H 7.39 (d, *J* = 7.4 Hz, 2H), 7.33 (app t, *J* = 7.6 Hz, 2H), 7.28 (d, *J* = 7.3 Hz, 1H), 6.65 (d, *J* = 15.6 Hz, 1H), 6.40 (dt, *J* = 15.6 & 7.8 Hz, 1H), 4.17 (d, *J* = 7.8 Hz, 2H); ^{13}C NMR (150 MHz, $CDCl_3$) δ_C 135.8 (*C*), 134.5 (*C*H), 128.6 (2 × *C*H), 128.3 (*C*H), 126.7 (2 × *C*H), 125.2 (*C*H), 33.4 (*C*H_2); IR (thin film) v: 3027.9 (w), 1645.9 (w), 1495.5 (w), 1449.9 (w), 1201.4 (m), 962.8 (s), 747.8 (s), 692.4 (s), 668.0 (m) cm^{-1}; HRMS *m*/*z* (EI$^+$): found 195.9887 ([M]$^+$, C_9H_9Br requires 195.9882), Δ = 2.6 ppm.

5-(Bromomethyl)-1,2,3-trimethoxybenzene (Entry 3) [5]

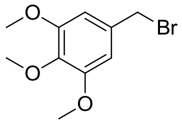

Isolated as an off-white amorphous solid (48.4 mg, 92% yield); ^{1}H NMR (600 MHz, $CDCl_3$) δ_H 6.61 (s, 2H), 4.46 (s, 2H), 3.87 (s, 6H), 3.84 (s, 3H); ^{13}C NMR (150 MHz, $CDCl_3$) δ_C 153.3 (2 × *C*), 138.1(*C*), 133.1 (*C*), 106.1 (2 × *C*H), 60.8 (*C*H_3), 56.1 (2 × *C*H_3), 34.2 (*C*H_2); IR (thin film) v: 2936.9 (w), 2836.8 (w), 1590.2 (m), 1504.7 (m), 1457.1 (m), 1420.1 (m), 1333.0 (m), 1239.7 (m), 1213.4 (m), 1115.5 (s), 1039.4 (w), 1004.2 (m), 910.6 (w), 831.2 (w), 780.7 (w), 671.7 (w) cm^{-1}; HRMS *m*/*z* (EI$^+$): found 260.0044 ([M]$^+$, $C_{10}H_{13}O_3Br$ requires 260.0043), Δ = 0.4 ppm.

1-(Bromomethyl)naphthalene (Entry 4) [6]

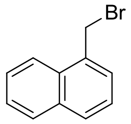

Isolated as a pale yellow oil (32.8 mg, 74% yield); ^{1}H NMR (600 MHz, $CDCl_3$) δ_H 8.17 (d, *J* = 8.5 Hz, 1H), 7.90 (d, *J* = 8.1 Hz, 1H), 7.85 (d, *J* = 8.2 Hz, 1H), 7.63 (dt, *J* = 7.6 & 0.8 Hz, 1H), 7.56–7.52 (m, 2H), 7.42 (app t, *J* = 7.6 Hz, 1H), 4.98 (s, 2H); ^{13}C NMR (150 MHz, $CDCl_3$) δ_C 134.0 (*C*), 133.2 (*C*), 131.0 (*C*), 129.8 (*C*H), 128.8 (*C*H), 127.7 (*C*H), 126.6 (*C*H), 126.2 (*C*H), 125.4 (*C*H) 123.7 (*C*H), 31.7 (*C*H_2); IR (thin film) v: 3047.1 (w), 1596.6 (w), 1510.3 (w), 1450.6 (w), 1398.3 (w), 1241.8 (w), 1206.7 (m), 1168.5 (w), 1016.9 (w), 866.0 (w), 798.8 (m), 773.9 (s), 734.1 (w), 706.7 (w) cm^{-1}; HRMS *m*/*z* (EI$^+$): found 219.9886 ([M]$^+$, $C_{11}H_9Br$ requires 219.9882), Δ = 1.8 ppm.

(1-Bromoethyl)benzene (Entry 5) [7]

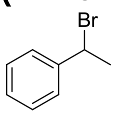

Isolated as a pale yellow oil (34.1 mg, 92% yield); ^{1}H NMR (600 MHz, $CDCl_3$) δ_H 7.44 (d, *J* = 7.6 Hz, 2H), 7.35 (app t, *J* = 7.6 Hz, 2H), 7.29 (t, *J* = 7.3 Hz, 1H), 5.22 (q, *J* = 6.9 Hz, 1H), 2.06 (d, *J* = 6.9 Hz, 3H); ^{13}C NMR (150 MHz, $CDCl_3$) δ_C 143.2 (*C*),

128.7 (2 × *C*H), 128.3 (*C*H), 126.8 (2 × *C*H), 49.5 (*C*H), 26.8 (*C*H_3); IR (thin film) ν: 3342.1 (w), 2961.2 (m), 2925.8 (m), 2855.0 (w), 1706.2 (w), 1687.8 (w), 1597.9 (w), 1492.2 (w), 1451.5 (m), 1377.8 (w), 1278.2 (m), 1201.7 (m), 1158.8 (m), 1058.1 (m), 1029.2 (m), 761.5 (m), 699.4 (s), 662.7 (w) cm^{-1}; HRMS *m/z* (EI^+): found 182.9804 ($[M]^+$, C_9H_9Br requires 182.9804), Δ = 0.1 ppm.

5-(Bromomethyl)benzo[*d*][1,3]dioxole (Entry 6) [8]

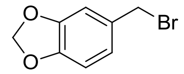

Isolated as a pale green solid (38.9 mg, 91%); ^1H NMR (600 MHz, $CDCl_3$) δ_H 6.88 (s, 1H), 6.86 (d, *J* = 8.4 Hz, 1H), 6.75 (d, *J* = 7.9 Hz, 1H), 5.97 (s, 2H), 4.46 (s, 2H); ^{13}C NMR (150 MHz, $CDCl_3$) δ_C 147.9 (*C*), 147.8 (*C*), 131.5 (*C*), 122.7 (*C*H), 109.5 (*C*H), 108.3 (*C*H), 101.3 (*C*H_2), 34.2 (*C*H_2); IR (thin film) ν: 2896.6 (w), 1608.0 (w), 1500.7 (m), 1487.7 (s), 1443.1 (s), 1360.4 (w), 1246.4 (s), 1191.1 (m), 1121.9 (w), 1095.5 (m), 1034.9 (s), 941.9 (w), 926.7 (s), 860.4 (w), 807.9 (m), 766.2 (m), 725.0 (w), 716.2 (w) cm^{-1}; HRMS *m/z* (EI^+): found 213.9629 ($[M]^+$, $C_8H_7O_2Br$ requires 213.9624), Δ = 2.3 ppm.

1-(Bromomethyl)-4-iodobenzene (Entry 7) [9]

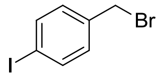

A single pass through the monolith gave 94% conversion by ^1H NMR. Recirculating through the monolith for 1 hour and 15 minutes gave the product as a pale yellow solid (56.1 mg, 95% yield); ^1H NMR (600 MHz, $CDCl_3$) δ_H 7.68 (d, *J* = 8.3 Hz, 2H), 7.13 (d, *J* = 8.3 Hz, 2H), 4.42 (s, 2H); ^{13}C NMR (150 MHz, $CDCl_3$) δ_C 137.9 (2 × *C*H), 137.4 (*C*), 130.8 (2 × *C*H), 94.1 (*C*), 32.5 (*C*H_2); IR (thin film) ν: 2922.7 (w), 2853.1 (w), 1579.7 (w), 1478.8 (m), 1395.9 (m), 1220.6 (m), 1201.4 (m), 1175.3 (w), 1087.5 (w), 1052.9 (m), 1004.6 (s), 956.6 (w), 872.5 (w), 824.8 (s), 795.7 (s), 710.8 (s) cm^{-1}; HRMS *m/z* (EI^+) found 295.8705 ($[M]^+$, C_7H_6BrI requires 295.8692), Δ = 4.2 ppm; GCMS Rt 14.98 min, *m/z* (EI^+): 298.0 ($C_7H_6{}^{81}BrI^{+\bullet}$, 5), 296.0 ($C_7H_6{}^{79}BrI^{+\bullet}$, 5), 218.2 ($C_7H_6I^{+\bullet}$, 7), 217.1 ($C_7H_5I^{+\bullet}$, 100).

During optimisation of the reaction for the above substrate, (4-iodophenyl)methanol was passed through the monolith whilst it was heated to 50 °C. Concentration of the output stream in vacuo gave a mixture of the starting material, desired 1-(bromomethyl)-4-iodobenzene and 1-(chloromethyl)-4-iodobenzene, determined to be a ratio of 1:11.1:5.1 (OH:Br:Cl) by ^1H NMR. The two halogenated products were inseparable by chromatography, but the raw data for the chloride was collected. The ^1H NMR data was found to match that of the literature [10], and the ^{13}C data was confirmed through HMQC analysis.

1-(Chloromethyl)-4-iodobenzene

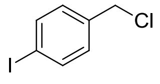

^1H NMR (600 MHz, $CDCl_3$) δ_H 7.67–7.70 (m, 2H), 7.13 (d, *J* = 8.2 Hz, 2H), 4.52 (s, 2H); ^{13}C NMR (150 MHz, $CDCl_3$) δ_C 138.0 (2 × *C*H), 137.6 (*C*), 130.6 (2 × *C*H), 94.3 (*C*), 45.6 (*C*H_2); HRMS *m/z* (EI^+): found 251.9201 ($[M]^+$, C_7H_6ClI requires 251.9197),

Δ = 1.6 ppm; GCMS Rt 13.86 min, *m/z* (EI$^+$) 254.0 ($C_7H_6{}^{37}ClI^{+\bullet}$, 11%), 252.1 ($C_7H_6{}^{35}ClI^{+\bullet}$, 39), 218.1 ($C_7H_6I^{+\bullet}$, 6), 217.1 ($C_7H_5I^{+\bullet}$, 100.00).

1-(Bromomethyl)-2-iodobenzene (Entry 8) [11]

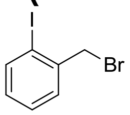

A single pass through the monolith gave 84% conversion by ^{1}H NMR. Recirculating through the monolith for 1 hour and 15 minutes gave the product as a pale yellow solid (56.3 mg, 95% yield); ^{1}H NMR (600 MHz, $CDCl_3$) δ_H 7.86 (d, *J* = 7.9 Hz, 1H), 7.48 (dd, *J* = 7.6 & 1.2 Hz, 1H), 7.34 (app t, *J* = 7.5 Hz, 1H), 6.98 (dd, *J* = 7.6 & 1.3 Hz, 1H), 4.60 (s, 2H); ^{13}C NMR (150 MHz, $CDCl_3$) δ_C 140.2 (*C*), 140.1 (*C*H), 130.5 (*C*H), 130.0 (*C*H), 128.8 (*C*H), 100.0 (*C*), 38.7 (CH_2); IR (thin film) ν: 1583.8 (m), 1560.8 (m), 1467.4 (m), 1435.8 (m), 1273.8 (m), 1217.1 (m), 1199.7 (m), 1044.3 (m), 1012.2 (s), 945.6 (m), 866.9 (m), 814.7 (m), 756.7 (s), 718.6 (s) cm^{-1}; HRMS *m/z* (EI$^+$): found 295.8701 ([M]$^+$, C_7H_6BrI requires 295.8692), Δ = 3.0 ppm.

***tert*-Butyl (2-(bromomethyl)phenyl)carbamate (Entry 9)** [12]

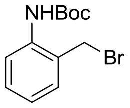

A single pass through the monolith gave 85% conversion by ^{1}H NMR. Recirculating through the monolith for 2 hour and 30 minutes gave the product as a clear glassy solid (38.9 mg, 68% yield); ^{1}H NMR (600 MHz, $CDCl_3$) δ_H 7.83 (d, *J* = 7.9 Hz, 1H), 7.32–7.35 (m, 1H), 7.28 (d, *J* = 7.5 Hz, 1H), 7.06 (app t, *J* = 7.4 Hz, 1H), 6.69 (br s, 1H), 4.51 (s, 2H), 1.54 (s, 9H); ^{13}C NMR (150 MHz, $CDCl_3$) δ_C 153.1(*C*), 137.2 (*C*H), 130.2 (2 × *C*H), 127.3 (*C*), 124.2 (*C*H), 123.0 (C), 81.1 (CH_2), 31.5 (*C*), 28.5 (3 × CH_3); IR (thin film) ν: 3104.4 (w), 2921.4 (w), 1716.2 (s), 1604.4 (w), 1499.7 (w), 1461.4 (w), 1420.0 (w), 1297.7 (w), 1266.2 (w), 1215.5 (w), 1065.4 (w), 888.1 (w), 746.7 (w) cm^{-1}; HRMS *m/z* (EI$^+$): found 285.0355 ([M]$^+$, $C_{12}H_{16}NO_2Br$ requires 285.0364), Δ = 3.2 ppm.

(4-Bromobutyl)benzene (Entry 10) [13]

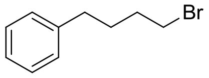

A single pass through the monolith gave <0.5% conversion by ^{1}H NMR. Recirculating through the monolith for 14 hours gave the product as a pale yellow oil (33.0 mg, 77% yield); ^{1}H NMR (600 MHz, $CDCl_3$) δ_H 7.29 (app t, *J* = 7.5 Hz, 2H), 7.18–7.21 (m, 3H), 3.43 (t, *J* = 6.7 Hz, 2H), 2.65 (t, *J* = 7.6 Hz, 2H), 1.88–1.93 (m, 2H), 1.76–1.81 (m, 2H); ^{13}C NMR (150 MHz, $CDCl_3$) δ_C 141.8 (*C*), 128.4 (4 × *C*H), 125.9 (*C*H), 35.0 (CH_2), 33.6 (CH_2), 32.2 (CH_2), 29.8 (CH_2); IR (thin film) ν: 2926.6 (w), 2856.3 (w), 1603.6 (w), 1495.9 (w), 1453.5 (w), 1250.7 (w), 1030.2 (w), 745.4 (m), 697.2 (s) cm^{-1}; HRMS *m/z* (EI$^+$): found 212.0186 ([M]$^+$, $C_{10}H_{13}Br$ requires 212.0195), Δ = 4.2 ppm.

References

1. Pangborn, A. B.; Giardello, M. A.; Grubbs, R. H.; Rosen, R. K.; Timmers, F. J. *Organometallics* **1996,** *15,* 1518–1520.
2. Gottlieb, H. E.; Kotlyar, V.; Nudelman, A. *J. Org. Chem.* **1997,** *62,* 7512–7515.
3. Altamura, M.; Perrotta, E. *J. Org. Chem.* **1993,** *58,* 272–274.
4. Mirjafaria, A.; Mohammadpoor-Baltork, I.; Moghadam, M.; Tangestaninejada, S.; Mirkhania, V.; Khosropoura, A. R. *Tetrahedron Lett.*, **2010**, *51*, 3274-3276.
5. Baumert, M.; Albrecht, M.; Winkler, H. D. F.; Schalley, C. A. *Synthesis* **2010,** *6,* 953–958.
6. Schönleber, M.; Hilgraf, R.; Pfaltz, A. *Adv. Synth. Catal.* **2008,** *350,* 2033–2038.
7. Guo, Z.-W.; Zamojski, A. *Tetrahedron* **1996,** *52,* 12553–12570.
8. Hirano, H.; Osawa, E.; Yamaoka, Y.; Yokoi, T. *Biol. Pharm. Bull.* **2001,** *24,* 1277–1281.
9. Vassilioua, S.; Xeilaria, M.; Yiotakisa, A.; Grembeckab, J.; Pawalczakc, M.; Kafarskib, P.; Muchab, A. *Bioorg. Med. Chem.* **2007,** *15,* 3187–3200.
10. Huang, Y.; Hammond, P. S.; Wu, L.; Mach, R. H. *J. Med Chem.* **2001,** *44,* 4404–4415.
11. Olivera, R.; SanMartin, R.; Domínguez, E.; Solans, X.; Urtiaga, M. K.; Arriortua, M. I. *J. Org. Chem.* **2000,** *65,* 6398–6411.
12. Mukai, C.; Kobayashi, M.; Kubota, S.; Takahashi, Y.; Kitagaki, S. *J. Org. Chem.* **2004,** *69,* 2128–2136.
13. Murphy, J. A.; Schoenebeck, F.; Findlay, N. J.; Thomson, D. W.; Zhou, S.; Garnier, J. *J. Am. Chem. Soc.* **2009,** *131,* 6475–6479.
